# Supplementary material for: Cross-species evolution of a highly potent AAV variant for therapeutic gene transfer and genome editing
Source: Nat Commun. 2022 Oct 10;13:5947. doi: 10.1038/s41467-022-33745-4 (PMC9548504; doi:10.1038/s41467-022-33745-4)
Supplement: Supplementary file 1 — Supplementary Information [file 41467_2022_33745_MOESM1_ESM.pdf]

Cross-species evolution of a highly potent AAV variant for therapeutic gene transfer and genome editing

Trevor J. Gonzalez[1], Katherine E. Simon[2,3#], Leo O. Blondel[2#], Marco M. Fanous[2], Angela L. Roger[4], Maribel Santiago Maysonet[5], Garth W. Devlin[2], Timothy J. Smith[1], Daniel K. Oh[2], L. Patrick Havlik[2], Ruth M. Castellanos Rivera[5], Jorge A. Piedrahita[3], Mai K. ElMallah[4], Charles A. Gersbach[6,7] and Aravind Asokan[1,2,6,7*]

Affiliations:

[1]Department of Molecular Genetics and Microbiology, Duke University School of Medicine; Durham, NC, USA.

[2]Department of Surgery, Duke University School of Medicine; Durham, NC, USA.

[3]North Carolina State University College of Veterinary Medicine; Raleigh, NC, USA.

[4]Department of Pediatrics, Duke University School of Medicine; Durham, NC, USA.

[5]StrideBio Inc.; Research Triangle Park, NC, USA.

[6]Duke Regeneration Center, Duke University School of Medicine; Durham, NC, USA.

[7]Department of Biomedical Engineering, Duke University; Durham, NC, USA.

# Contributed equally

* Corresponding Author: Aravind Asokan, Ph.D., 5148, MSRB3, 3 Genome Court, Durham NC 27710, USA. Email: aravind.asokan@duke.edu

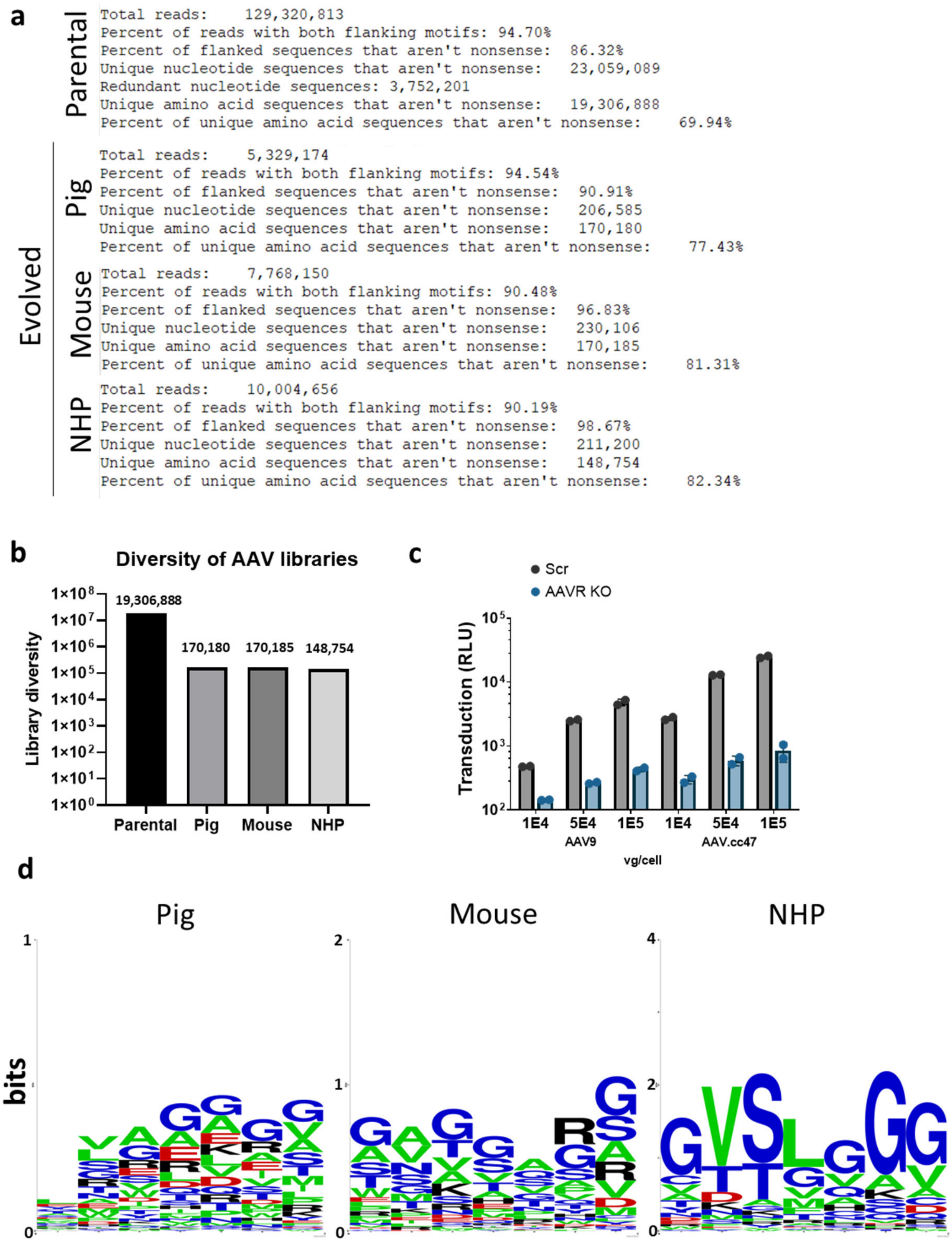

Supplementary Figure 1. Additional next generation sequencing (NGS) analyses from AAV capsid libraries and assessment of AAVR dependence. (a) Library diversity was determined using a custom in-house Perl script. Reads were surveyed for nucleotide

sequences flanking the library regions and intermediate sequences were counted and ranked. These nucleotide sequences were then translated and the resulting amino acid sequences were also counted and ranked. (**b**) GraphPad prism was used to visualize the library diversity in bar graphs and total unique amino acid sequences are listed above each bar. (**c**) Transduction of HuH7 scramble and AAVR knockout cell lines by AAV9 and evolved variant AAV.cc47 packaging a single stranded CBA-luciferase genome. Data in panel C are from two biological replicates. (**d**) Consensus motif analysis of the top 100 enriched AAV mutants after each round of evolution in pigs, mice, and NHPs, for residues 452 - 458 (VP1 numbering). Source data are provided as a Source Data file.

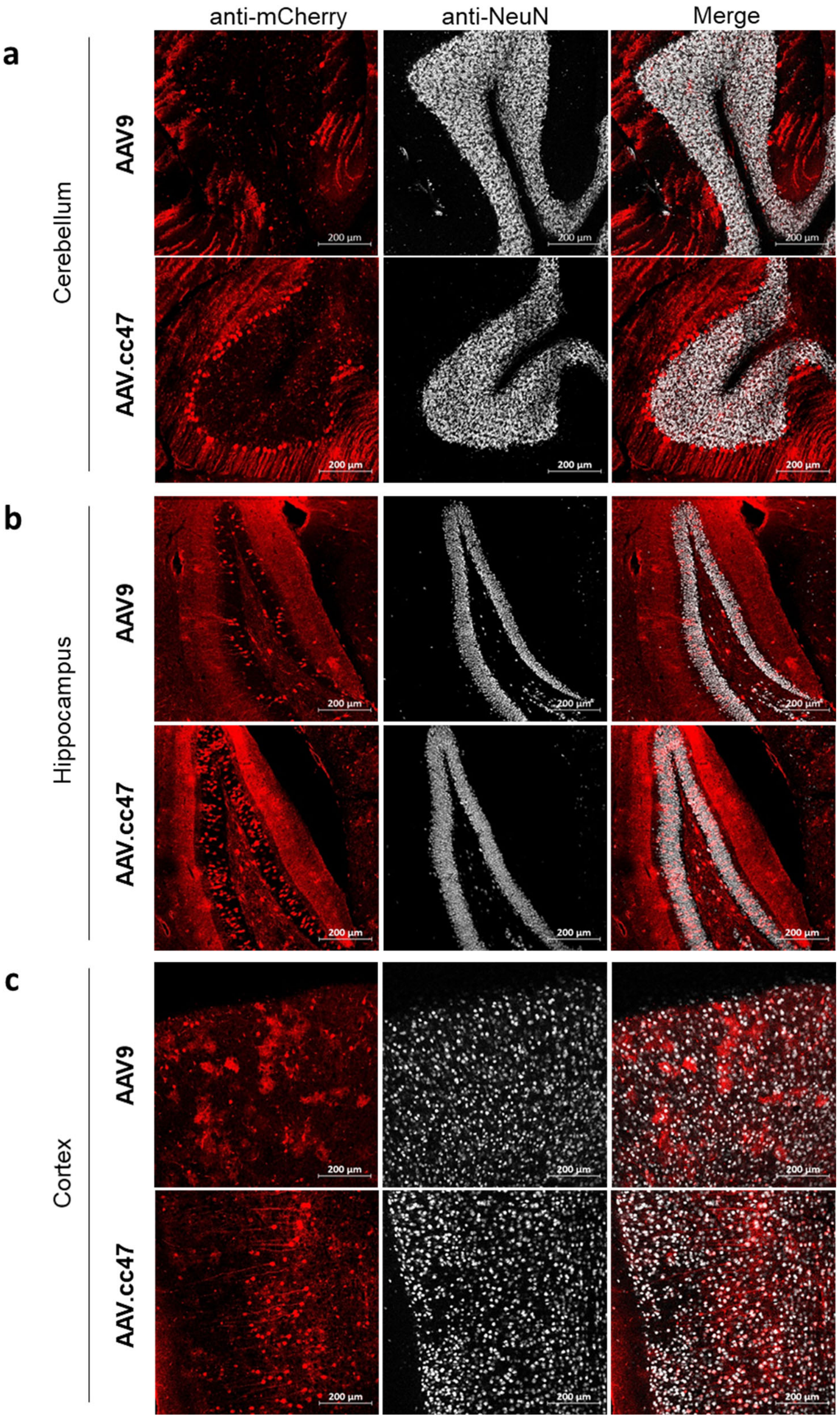

Supplementary Figure 2. Representative immunofluorescence images for mCherry and NeuN proteins in C57/B6 brain used for percent transduction quantification in neurons.

8-10 week old C57/B6 mice (n=3) were injected intravenously at a dose of 5e13vg/kg with

AAV9 or AAV.cc47. Vectors delivered a self-complementary AAV genome with the chicken-beta actin hybrid (CBh) promoter driving mCherry reporter expression. Mice were sacrificed 4 weeks post injection and whole brains were sectioned 50µm thick with a vibratome. Representative immunofluorescence images used in quantification are shown for cerebellum (**a**), hippocampus (**b**), and cortex (**c**). mCherry protein (red) NeuN protein (white).

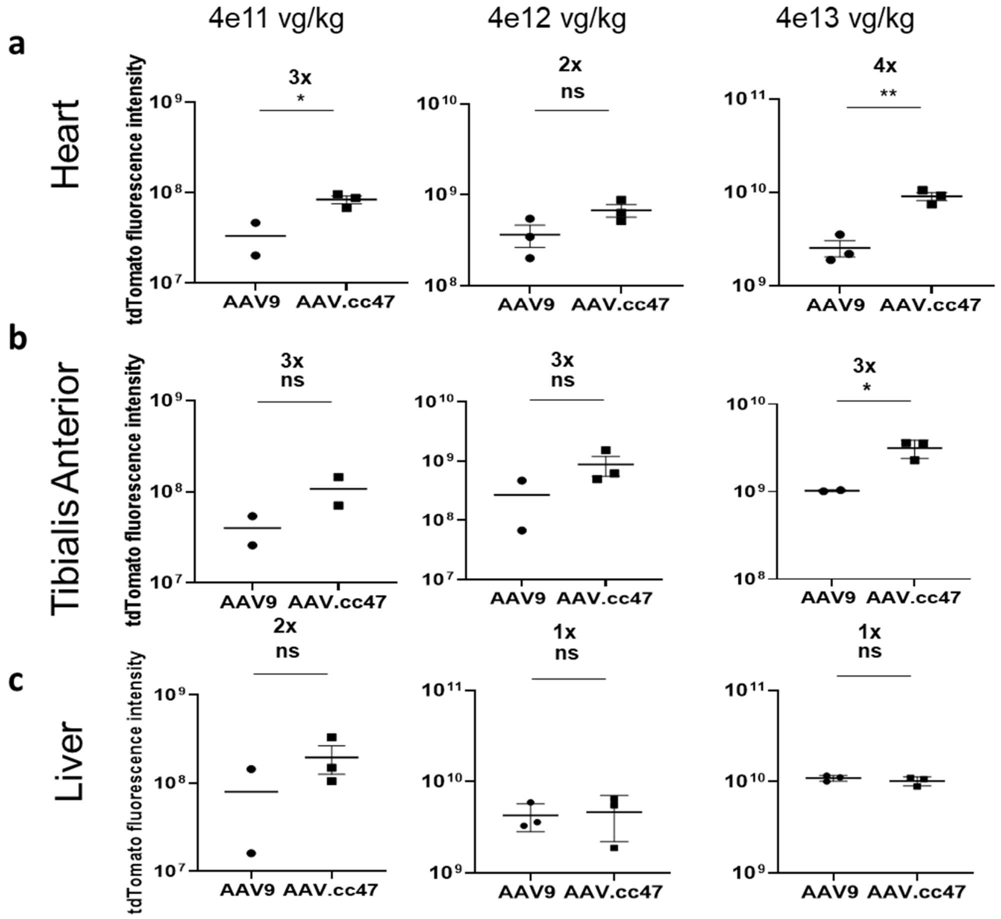

Supplementary Figure 3. tdTomato fluorescence intensity quantification in Ai9 heart, skeletal muscle, and liver. 8-10 week old Ai9 mice (n=3) injected systemically with a CMV-Cre expressing AAV9 or AAV.cc47 vector. tdTomato fluorescence intensity was

calculated from tissue stained for tdTomato protein by immunofluorescence in heart (**a**), skeletal muscle (**b**), and liver (**c**). The vectors were dosed at 4e11, 4e12, and 4e13 vg/kg. Each dot represents an individual mouse, fold change is listed above significance, the dash represents the mean value and error bars represent the standard error mean. Statistical significance was determined by an unpaired two-tailed Student *t* Test (High dose heart, $P < 0.0031$; tibialis anterior $P < 0.0309$; liver $P < 0.3890$; Mid dose heart, $P < 0.1003$; tibialis anterior, $P < 0.2637$; liver, $P < 0.8328$; low dose heart, $P < 0.0403$; tibialis anterior, $P < 0.2295$; liver, $P < 0.3374$). *$P < 0.05$; **$P < 0.01$; ns not significant. Source data are provided as a Source Data file.

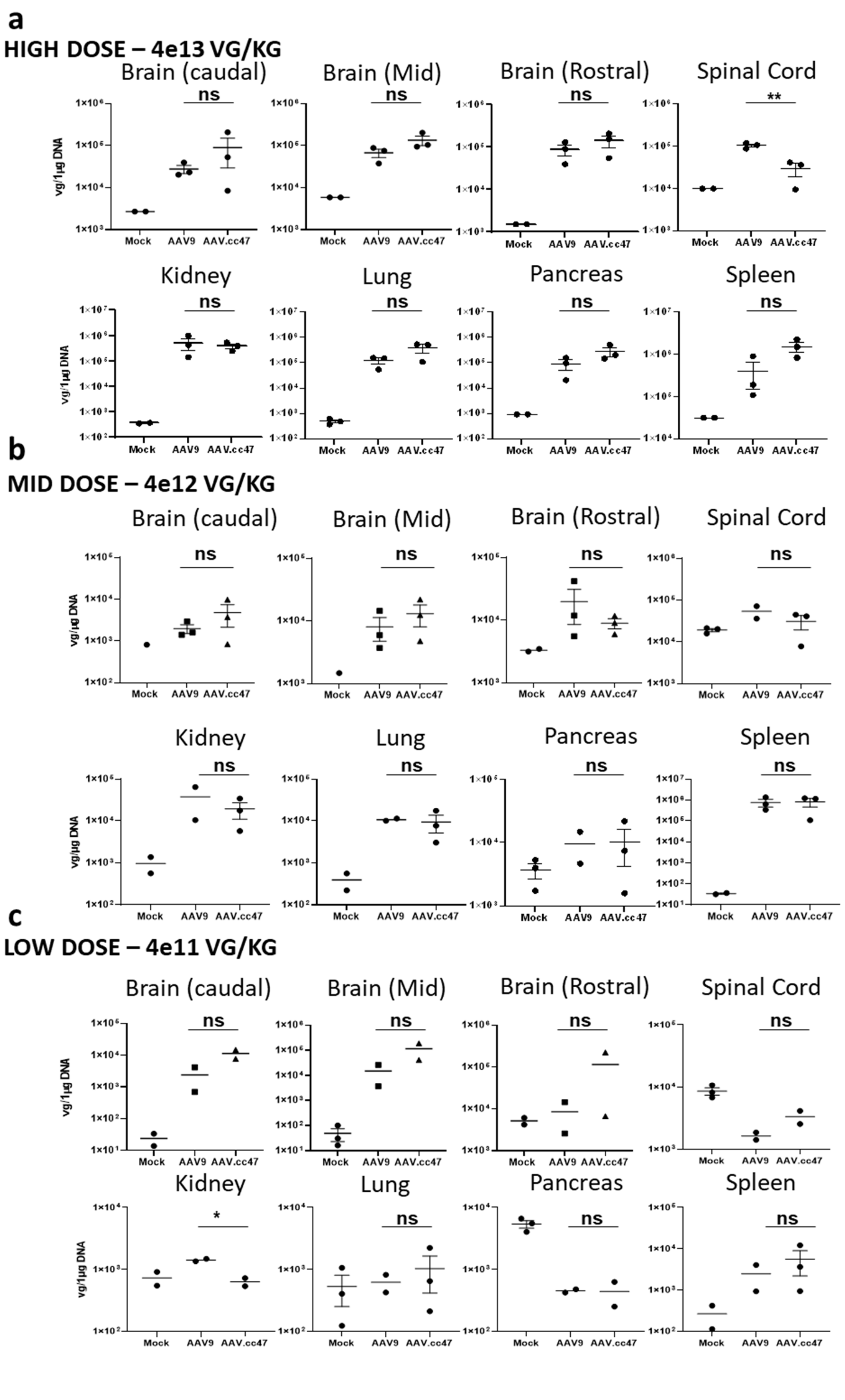

Supplementary Figure 4. Other organ vector genome biodistribution following systemic administration of Cre recombinase at 4e11, 4e12, and 4e13vg/kg in Ai9 mice. (**a-c**) 8-10

week old Ai9 mice (n=3) were used for each dose to calculate vector genome (vg) copy numbers per μg DNA. This was calculated by normalizing Cre recombinase copy numbers to total μg DNA input for qPCR quantification and are represented as log vg/μg DNA. Each dot represents an individual mouse, the dash represents the mean value and error bars represent the standard error mean. Statistical significance was determined by One-Way ANOVA with Tukey's posttest (High dose brain caudal, $P < 0.5318$, midbrain, P < 0.2129, rostral, $P < 0.5415$; spinal cord $P < 0.0034$; kidney, $P < 0.8635$; lung, $P < 0.1357$; pancreas $P < 0.2549$; spleen, $P < 0.1013$; Mid dose brain caudal, $P < 0.5918$, midbrain, $P < 0.7029$, rostral, $P < 0.5712$; spinal cord, $P < 0.3847$; kidney, $P < 0.6531$; lung, $P < 0.9580$; pancreas, $P < 0.9962$; spleen, $P < 0.9900$; low dose brain caudal, $P < 0.1392$; midbrain, $P < 0.2535$; rostral, $P < 0.5285$; spinal cord, $P < 0.5588$; kidney, $P < 0.0417$; lung, $P < 0.8270$; pancreas, *P* < 0..9999; spleen, $P < 0.7276$). *$P < 0.05$; **$P < 0.01$; ns not significant. Source data are provided as a Source Data file.

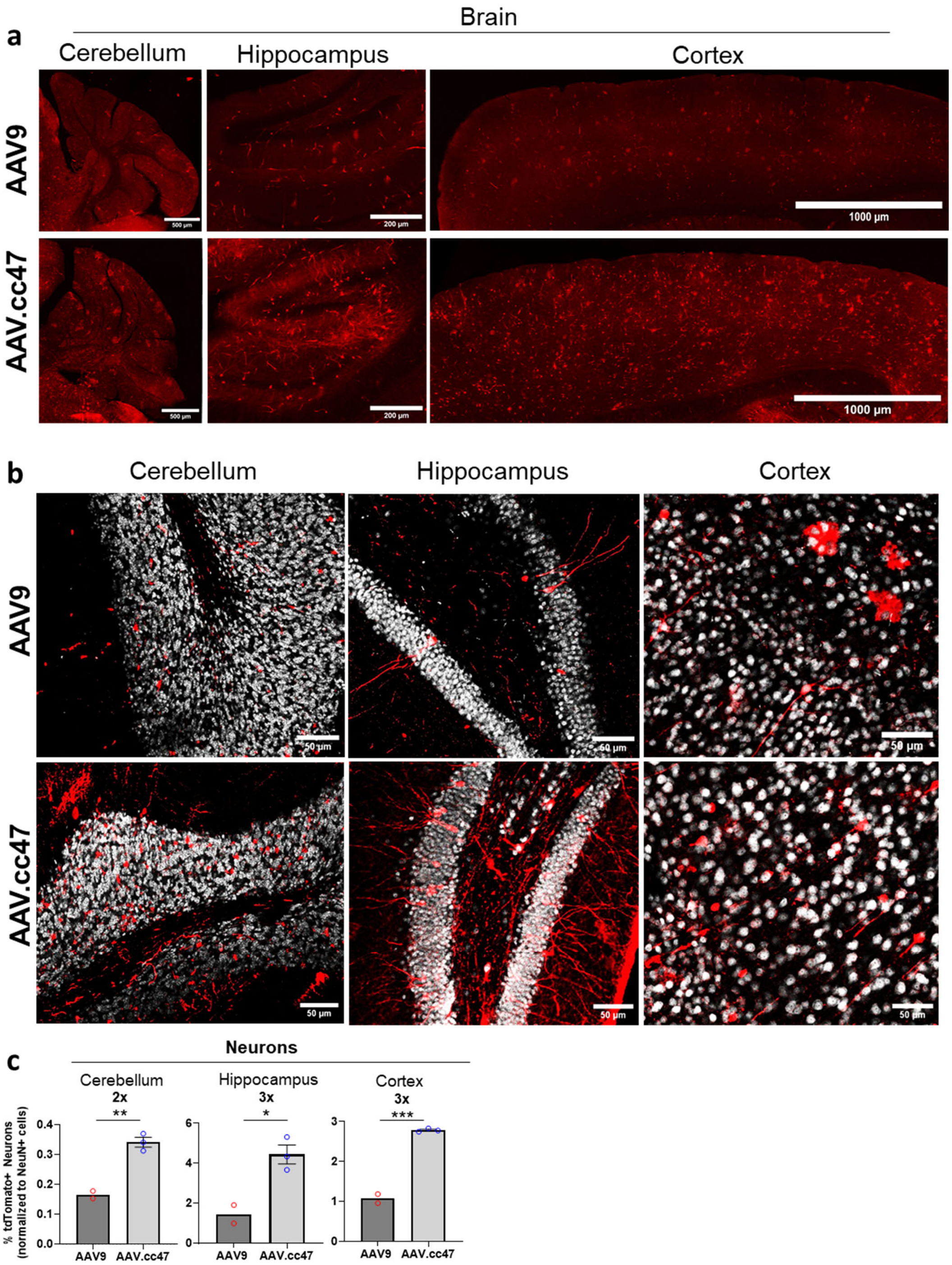

Supplementary Figure 5. AAV.cc47 transduces Ai9 brain more effectively than AAV9.

8-10 week old Ai9 mice (n=3) were injected systemically with 4e13 vg/kg of AAV.cc47 or AAV9 vectors packaging a single stranded genome with a CMV driven Cre recombinase transgene. Mice were sacrificed 4 weeks post injection and whole brains were sectioned

50μm thick with a vibratome. (**a**) Representative immunofluorescence images of tdTomato in cerebellum, hippocampus, cortex, and whole brain. (**b**) Tissue sections were stained against tdTomato (red) and NeuN (white) proteins via immunofluorescence for co-staining quantification. (**c**) Quantification of total % transduced neurons in Ai9 brain by AAV9 and AAV.cc47 was determined by taking the ratio of the total tdTomato+ neurons compared to the total NeuN+ neurons in each brain region shown. For histological quantification, 2 sections per mouse and 3 images per section (total of 6 images) were used to quantify % transduced neurons using ImageJ. Each dot represents an individual mouse, fold change is listed above significance, the bars and graphs represent the mean value and error bars represent the standard error mean. Statistical significance was determined by an unpaired two-tailed Student t Test (cerebellum, $P < 0.0048$; hippocampus, $P < 0.0240$; cortex, $P < 0.0003$). *$P < 0.05$; **$P < 0.01$; ***$P < 0.001$; ns not significant. Source data are provided as a Source Data file.

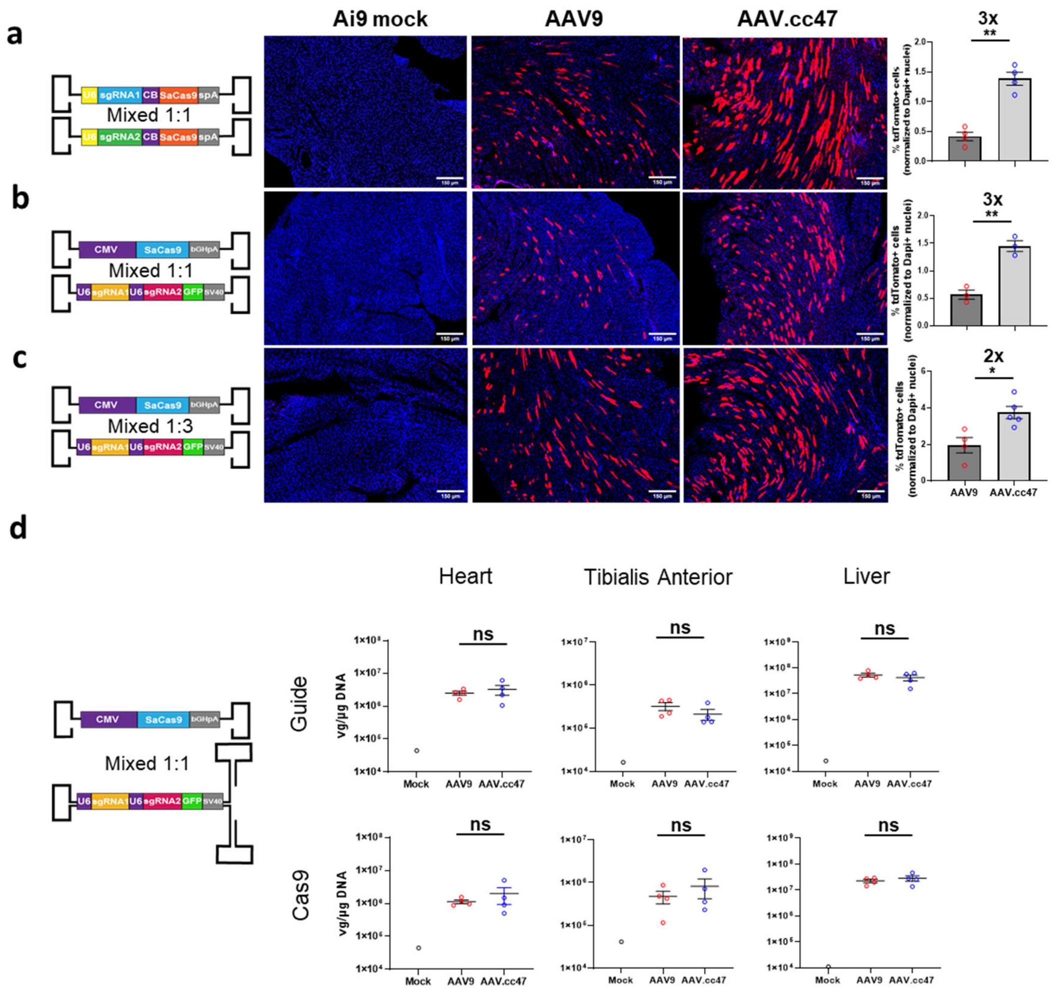

Supplementary Figure 6. Other AAV-CRISPR strategies evaluated in Ai9 heart and vector genome biodistribution quantification for our lead AAV-CRISPR strategy. 8-10 week old Ai9 mice (n=4) were injected systemically with different dual AAV-CRISPR cassettes packaged into AAV9 (red) and AAV.cc47 (blue). These dual vectors include two single stranded AAV genomes delivering 1 U6 driven gRNA each targeting the *Rosa26* locus plus CB driven SaCas9 on each vector (**a**); two single stranded AAV genomes with one delivering CMV driven SaCas9 and the other delivering 2 gRNAs targeting the *Rosa26* locus driven by U6 promoters (**b**); and the same design as in panel B but instead the gRNA vector was increased in the total preparation to reflect a 1 to 3 cas9 to gRNA ratio (**c**). The total doses administered systemically were 1.0e14, 1.5e14, and 2.0e14 vg/kg, respectively. 50µm

thick cross sections were obtained via a vibratome for heart. Quantification of the percentage of tdTomato+ cells was normalized to the number of DAPI+ nuclei. For all histological quantification, 2 sections per mouse and 3 images per section (total of 6 images) were used to quantify % transduced cells using ImageJ. Vector genome (vg) copy numbers per µg DNA were calculated in Ai9 heart, tibialis anterior, and liver with our lead strategy consisting of a single stranded AAV genome delivering CMV driven SaCas9 plus a self-complementary genome delivering 2 gRNAs targeting the *Rosa26* locus driven by U6 promoters (**d**). CMV (SaCas9 vector) and eGFP (gRNA vector) copy numbers were normalized to total µg DNA input for qPCR quantification and are represented as log vg/µg DNA. The total dose administered systemically was 2.0e14 vg/kg. Each dot represents an individual mouse, the dash represents the mean value and error bars represent the standard error mean. Statistical significance was determined by One-Way ANOVA with Tukey's posttest (3 treatment groups) or an unpaired two-tailed Student *t* Test (2 treatment groups; CB single stranded, $P < 0.0003$; CMV 1:1, $P < 0.0024$; CMV 1:3, $P < 0.0120$). *$P < 0.05$; **$P < 0.01$; ns not significant. Source data are provided as a Source Data file.

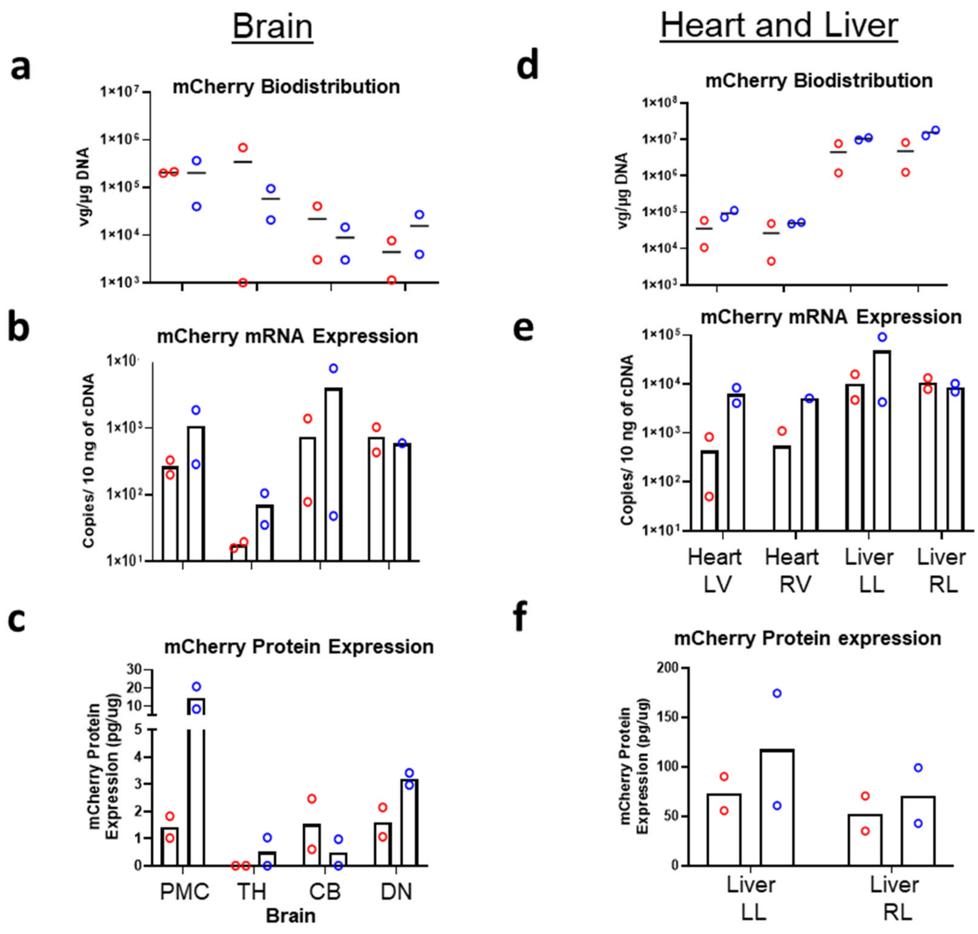

Supplementary Figure 7. Quantification of mCherry DNA, RNA, and protein levels in non-human primate (NHP) brain, heart, and liver following ICM infusion of AAV9 or AAV.cc47. NHPs were infused ICM with 1e13 total vg of AAV9 (red) or AAV.cc47 (blue) vectors in 2-year-old cynomolgus macaques (n=2). AAV vector genomes (**a**) in the brain were quantified via quantitative PCR with primers against the mCherry transgene. Vector genome copy numbers per µg DNA were calculated by normalizing mCherry recombinase copy numbers to total µg DNA input for qPCR quantification and are represented as log vg/µg DNA. mCherry mRNA expression (**b)** in the brain was calculated via quantitative RT-PCR and normalizing mCherry mRNA copy numbers to 10ng cDNA input used for qPCR quantification. Data is represented as log mRNA copies/10ng of cDNA. Quantification of mCherry protein expression (**c**) in the brain was calculated by normalizing mCherry protein

levels to μg protein input for ELISA quantification. Data is represented as mCherry protein expression in picograms per μg protein input. Quantification of vector genome biodistribution (**d),** mCherry mRNA (**e**), and mCherry protein (**f**) expression in liver and/or heart was also performed. AAV9 and AAV.cc47 treated NHPs are shown in red or blue dots, respectively. NHP brains were partitioned into premotor cortex (PMC), thalamus (TH), cerebellum (CB), dentate nucleus (DN), hearts were partitioned into left (LV) and right (RV) ventricles, and livers were partitioned into left (LL) and right (RL) lobes prior to analysis. Multiple replicate samples were taken from each region prior to DNA, RNA, and protein extraction and were used for subsequent quantitative PCR, quantitative RT-PCR, or ELISA analyses. Each dot represents an individual NHP and the dashes and bars represent the mean. Source data are provided as a Source Data file.

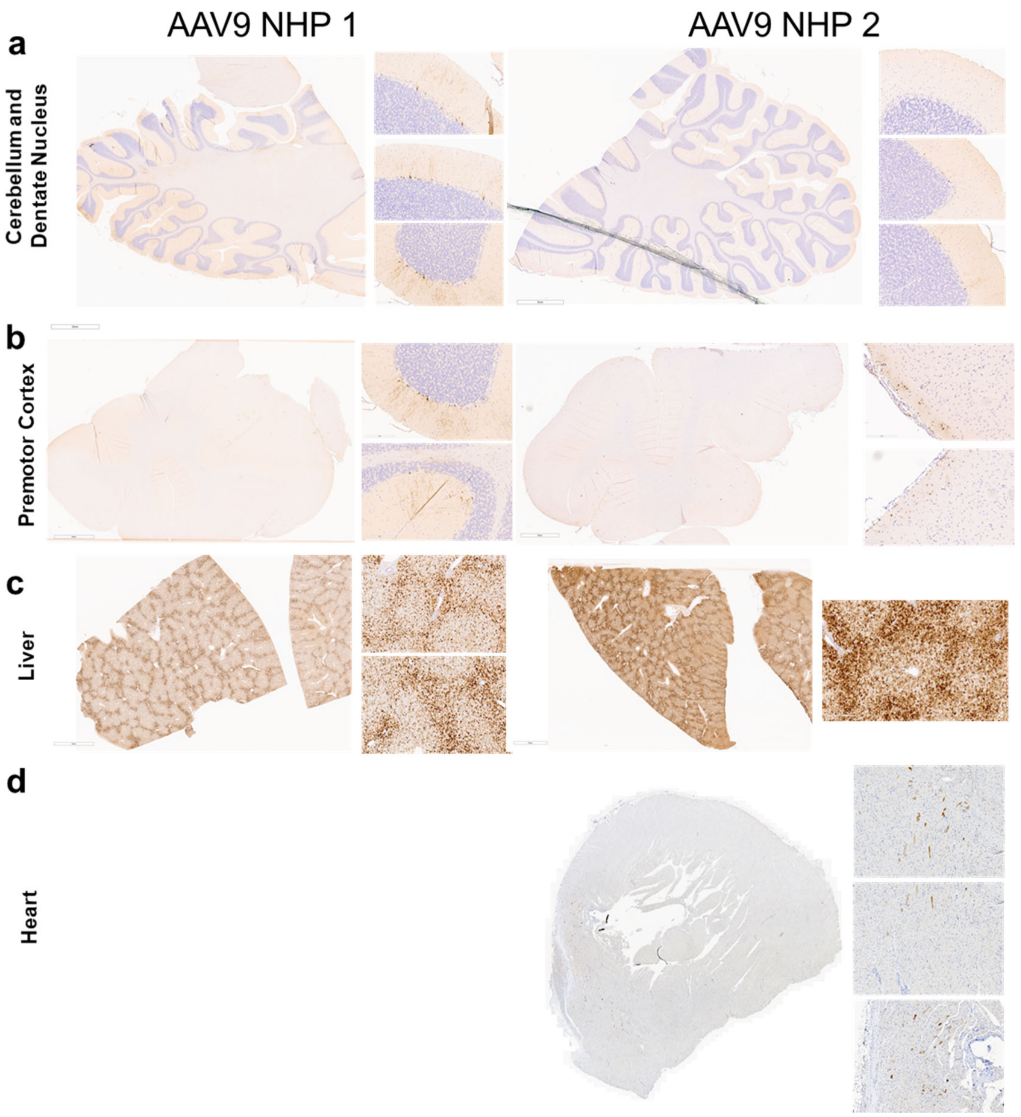

Supplementary Figure 8. Additional images from each NHP infused ICM with AAV9 vectors. NHPs were infused ICM with 1e13 total vg of AAV9 or AAV.cc47 vectors in 2-year-old cynomolgus macaques (n=2). Vectors packaged a self-complementary AAV genome with the CBh promoter driving a mCherry transgene. Immunohistochemistry was performed against mCherry protein in NHP cerebellum and dentate nucleus (**a**), premotor cortex (**b**), liver (**c**), and heart (**d**). Tissue was embedded in paraffin following fixation and cut 5µm thick. An anti-mCherry primary antibody was incubated on tissue and detected via a Vectastain ABC-HRP kit and mCherry expression was visualized with 3,3′-

Diaminobenzidine (DAB) in tissues. The in-life portion of this study was only performed once. The histological staining was repeated independently with similar results.

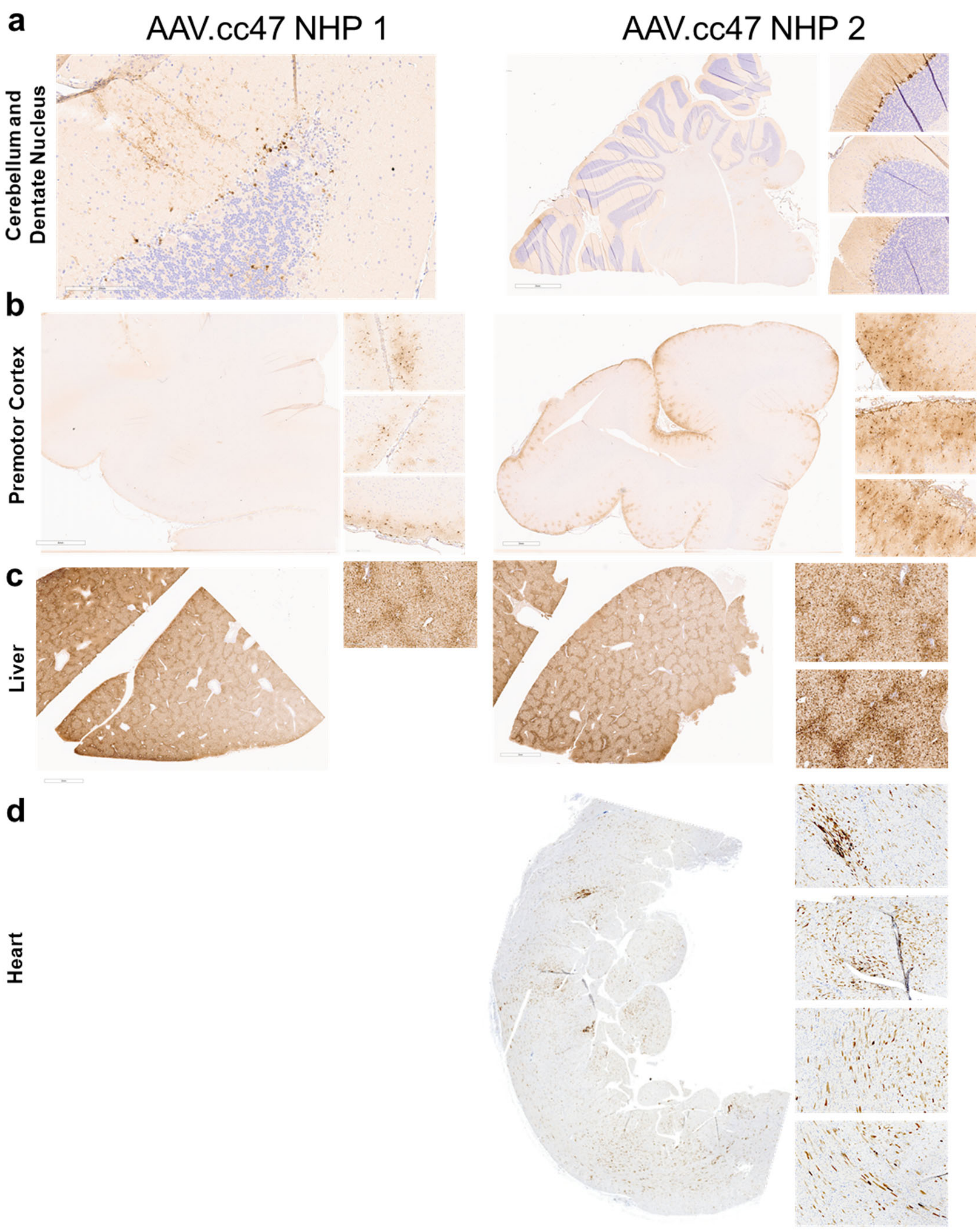

Supplementary Figure 9. Additional images from each NHP infused ICM with AAV.cc47 vectors. NHPs were infused ICM with 1e13 total vg of AAV9 or AAV.cc47 vectors in 2-year-old cynomolgus macaques (n=2). Vectors packaged a self-complementary AAV genome with the CBh promoter driving a mCherry transgene. Immunohistochemistry was performed against mCherry protein in NHP cerebellum and dentate nucleus (**a**), premotor cortex (**b**), liver (**c**), and heart (**d**). Tissue was embedded in paraffin following

fixation and cut 5µm thick. An anti-mCherry primary antibody was incubated on tissue and detected via a Vectastain ABC-HRP kit and mCherry expression was visualized with 3,3′-Diaminobenzidine (DAB) in tissues. The in-life portion of this study was only performed once. The histological staining was repeated independently with similar results.

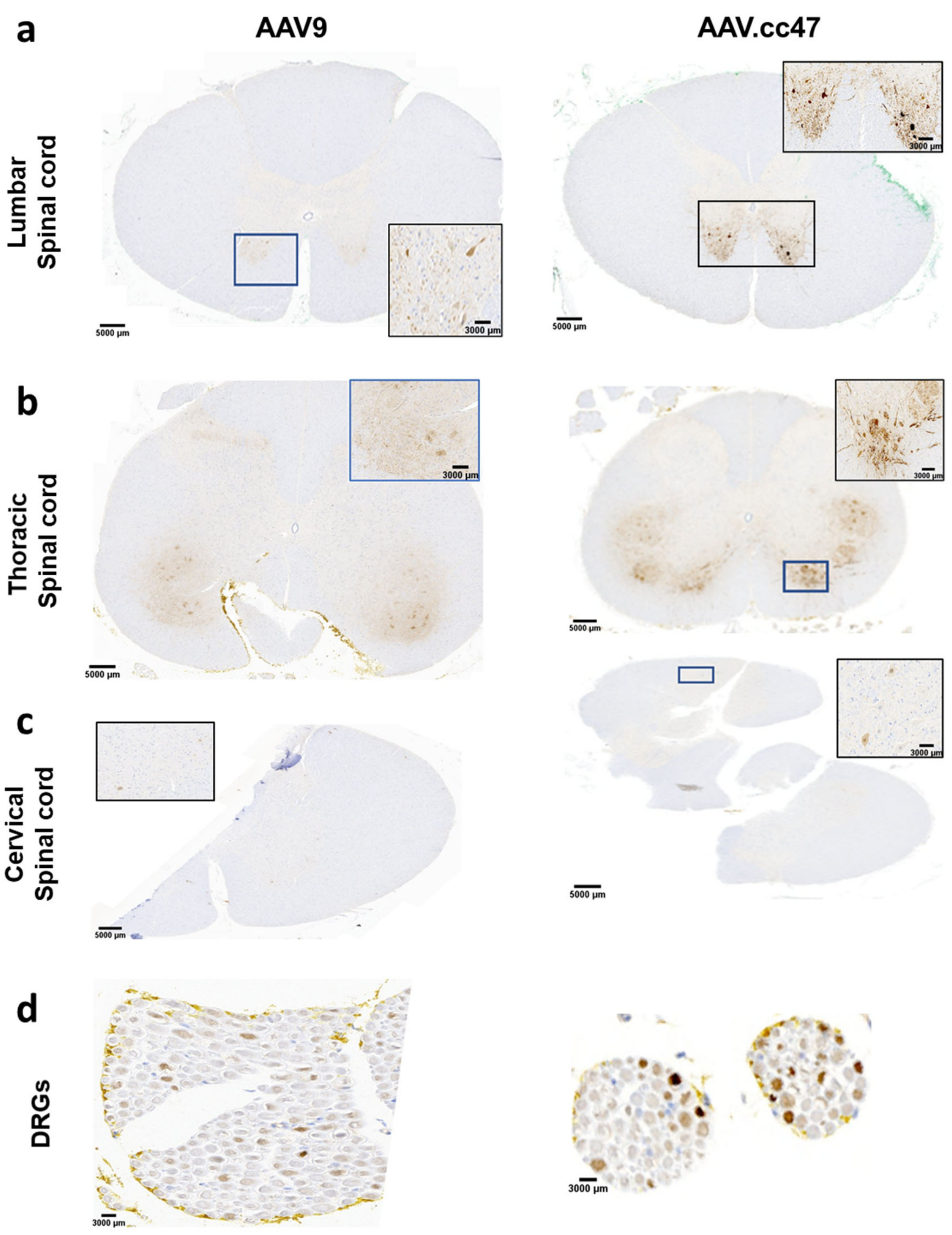

Supplementary Figure 10. Representative images from spinal cord and dorsal root ganglia (DRG) from NHPs infused ICM with AAV9 or AAV.cc47 vectors. NHPs were infused ICM with 1e13 total vg of AAV9 or AAV.cc47 vectors in 2-year-old cynomolgus macaques (n=2). Vectors packaged a self-complementary AAV genome with the CBh promoter driving a mCherry transgene. Immunohistochemistry was performed against mCherry protein in NHP lumbar (**a**), thoracic (**b**), cervical (**c**) spinal cords, and DRGs (**d**). Tissue was embedded in paraffin following fixation and cut 5μm thick. An anti-mCherry primary antibody was incubated on tissue and detected via a Vectastain ABC-HRP kit and mCherry expression was visualized with 3,3′-Diaminobenzidine (DAB) in tissues. The in-life portion of this study was only performed once. The histological staining was repeated independently with similar results.

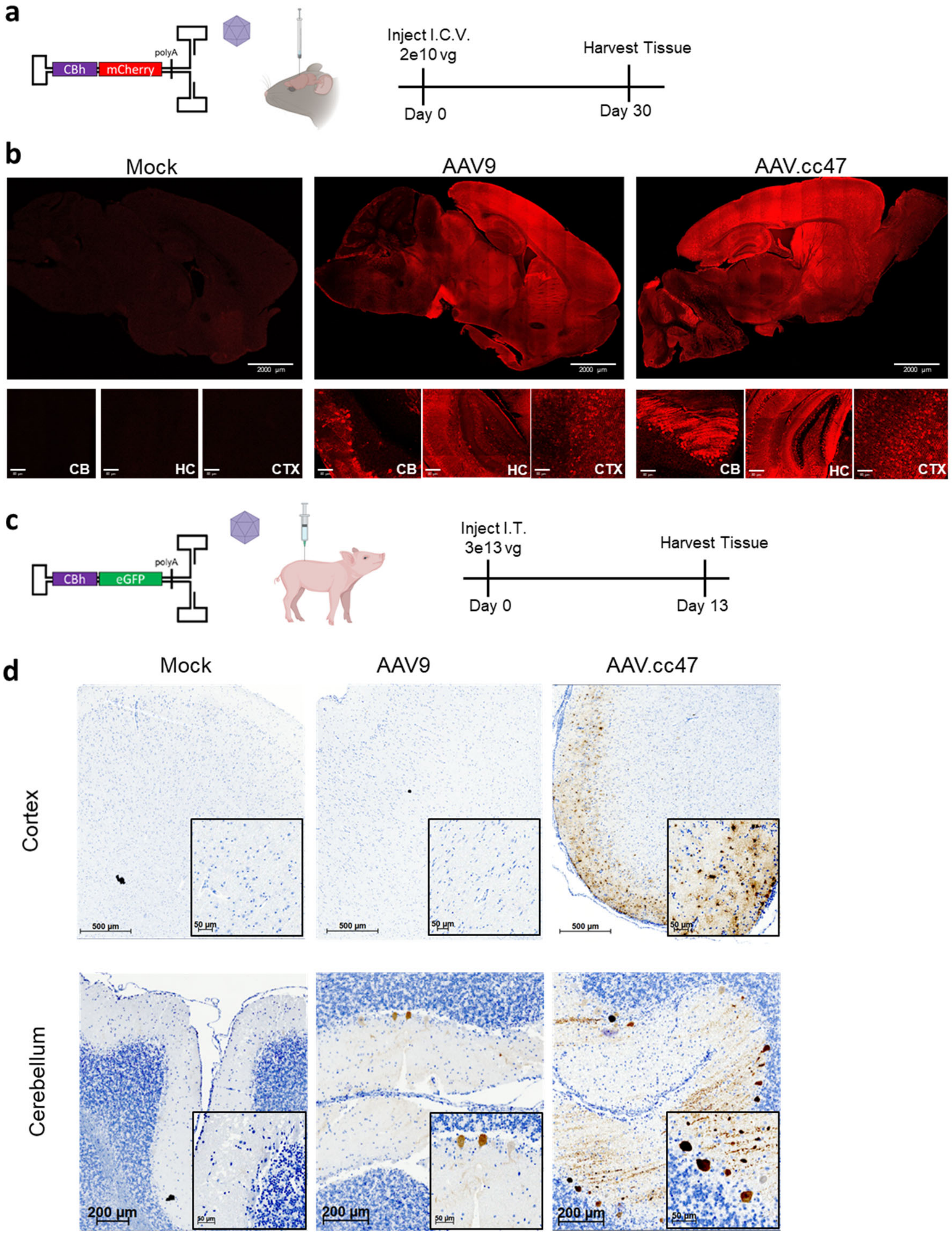

Supplementary Figure 11. IntraCSF dosing of AAV.cc47 or AAV9 following intracerebroventricular (ICV) injection in mice and intrathecal (IT) infusion in pigs. (a) P0-P1 C57/B6 neonates were injected ICV at a total dose of 2e10vg with AAV9 or AAV.cc47 and brains were harvested 4 weeks post injection. Vectors delivered a self-

complementary AAV genome with the CBh promoter driving mCherry expression. (**b**) Representative immunofluorescence images of mCherry in whole brain, cerebellum (CB), hippocampus (HC), and cortex (CTX). 50μm thick cross sections were obtained via a vibratome. An anti-mCherry primary antibody was incubated on tissue and detected with an AlexaFluor 647 secondary antibody. (**c**) Newly weaned piglets 3 weeks of age were administered a total of 3e13vg with AAV9 or AAV.cc47 via intrathecal infusion into the lumbar cistern and sacrificed 13 days post infusion. Vectors delivered a self-complementary AAV genome with the CBh promoter driving GFP expression. (**d**) Representative images of immunohistochemistry against eGFP in pig brain cortex (CTX) and cerebellum (CB). Tissue was embedded in paraffin following fixation and cut 5μm thick. An anti-eGFP primary antibody was incubated on tissue and detected via a Vectastain ABC-HRP kit and eGFP expression was visualized with 3,3′-Diaminobenzidine (DAB) in tissue. The in-life portions in mice were repeated independently with similar results. The in-life portions in pigs were only performed once. The histological staining was repeated independently with similar results for both mice and pigs.

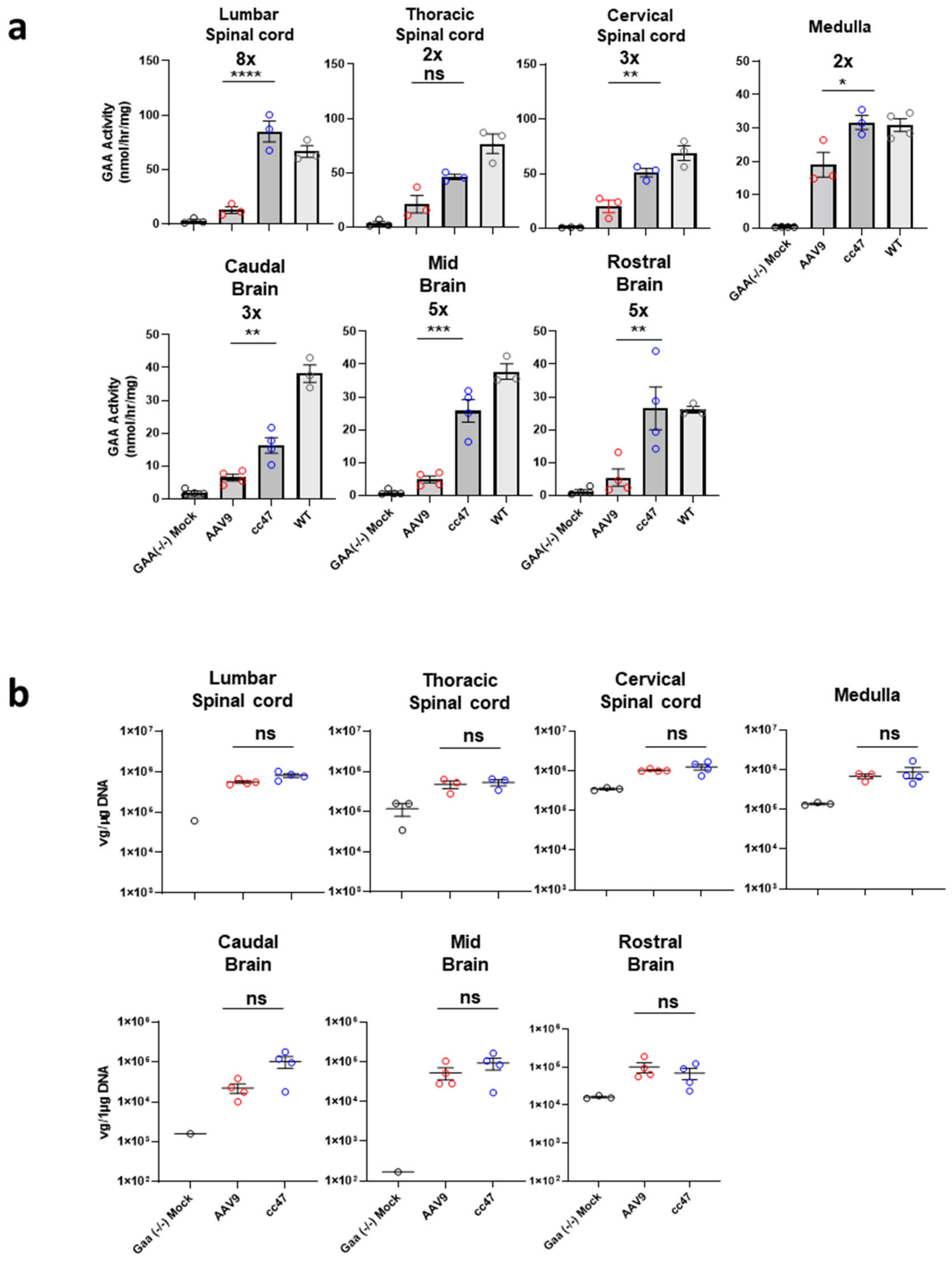

Supplementary Figure 12. Additional GAA enzyme levels and vector genome biodistribution in the *Gaa* (-/-) mouse model of Pompe disease. 8-10 week old *Gaa* (-/-) mice (n=4) were injected systemically with 1.3e14vg/kg of a single stranded AAV genome expressing the human acid alpha-glucosidase gene driven by the CBh promoter. Mice were

sacrificed 4 weeks post injection and tissue were harvested for analysis **(a)** GAA enzyme activity was quantified in the CNS of AAV9 (red) or AAV.cc47 (blue) treated *Gaa* (-/-) mice, untreated wild-type mice (grey), and untreated *Gaa* (-/-) mice (black). Data is represented as nmol per hour activity per mg BSA protein. (**b**) Vector genome copy numbers per μg DNA was calculated by normalizing SV40 polyA copy numbers to total μg DNA input for qPCR quantification and are represented as log vg/μg DNA. Mouse brains were partitioned into rostral, midbrain, and caudal regions and spinal cord was partitioned in lumbar, thoracic, lumbar regions prior to DNA extraction. Each dot represents an individual *Gaa* (-/-) mouse, fold change is listed above significance, the bars and dashes represent the mean GAA activity and vector genome biodistribution, respectively, of all mice analyzed and error bars represent the standard error mean. Statistical significance was determined by One-Way ANOVA with Tukey's posttest (GAA enzyme lumbar spinal cord, $P < 0.0001$, thoracic spinal cord, $P < 0.0002$, cervical spinal cord, $P < 0.0083$, medulla, P < 0.0116; brain caudal, $P < 0.0080$, midbrain, $P < 0.0001$, rostral, $P < 0.0091$). *$P < 0.05$; **$P < 0.01$; ***$P < 0.001$; ****$P < 0.0001$; ns not significant. Source data are provided as a Source Data file.

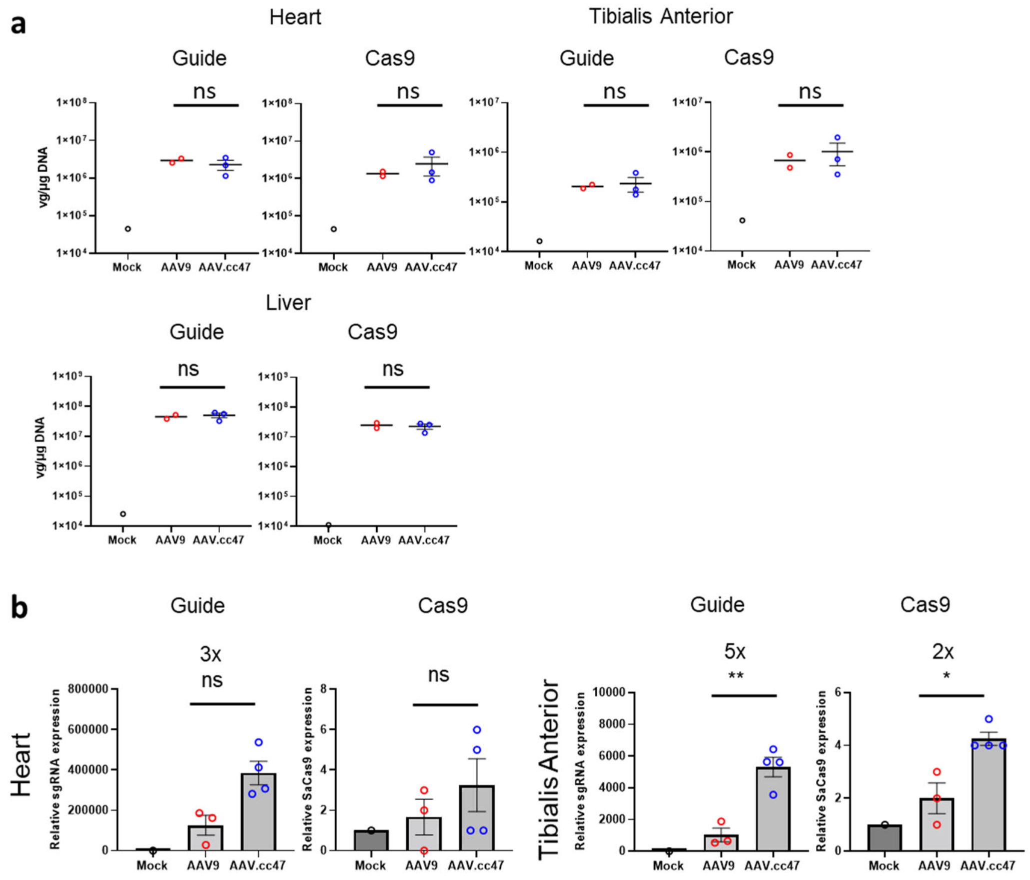

Supplementary Figure 13. Quantification of gRNA expression, SaCas9 mRNA, and vector genome biodistribution in mdx heart and tibialis anterior following IV dosing of our lead self-complementary AAV-CRISPR strategy. 8-10 week old Pax7-nGFP mdx mice (n=4) were injected systemically with AAV9 (red) or AAV.cc47 (blue) vectors. A total dose of 2.8e14 vg/kg was administered consisting of a single stranded genome expressing SaCas9 driven by the CMV promoter and a self-complementary genome expressing two gRNAs driven by the U6 promoter targeting exon 23 of *Dmd,* mixed in a 1:1 ratio. Mice were sacrificed 4 weeks post injection. **(a)** Vector genome copy numbers per µg DNA was determined via quantitative PCR and CMV (SaCas9) and eGFP (gRNA) copy numbers were normalized to total µg DNA input for qPCR quantification. Data is represented as log vg/µg DNA in mdx heart, tibialis anterior, and liver. (**b**) Quantitative RT-PCR was performed with

primers amplifying each gRNA or SaCas9 mRNA. RNA levels were normalized to mouse *Actb* housekeeping gene. Each dot represents an individual mouse, fold change is listed above each bar graph, the dashes and bars represent the mean value and error bars represent the standard error mean. Statistical significance was determined by One-Way ANOVA with Tukey's posttest (qRT-PCR heart gRNA, $P < 0.0514$, Cas9, $P < 0.6527$; tibialis anterior gRNA, $P < 0.0074$, Cas9, $P < 0.0240$). *$P < 0.05$; **$P < 0.01$; ns not significant. Source data are provided as a Source Data file.

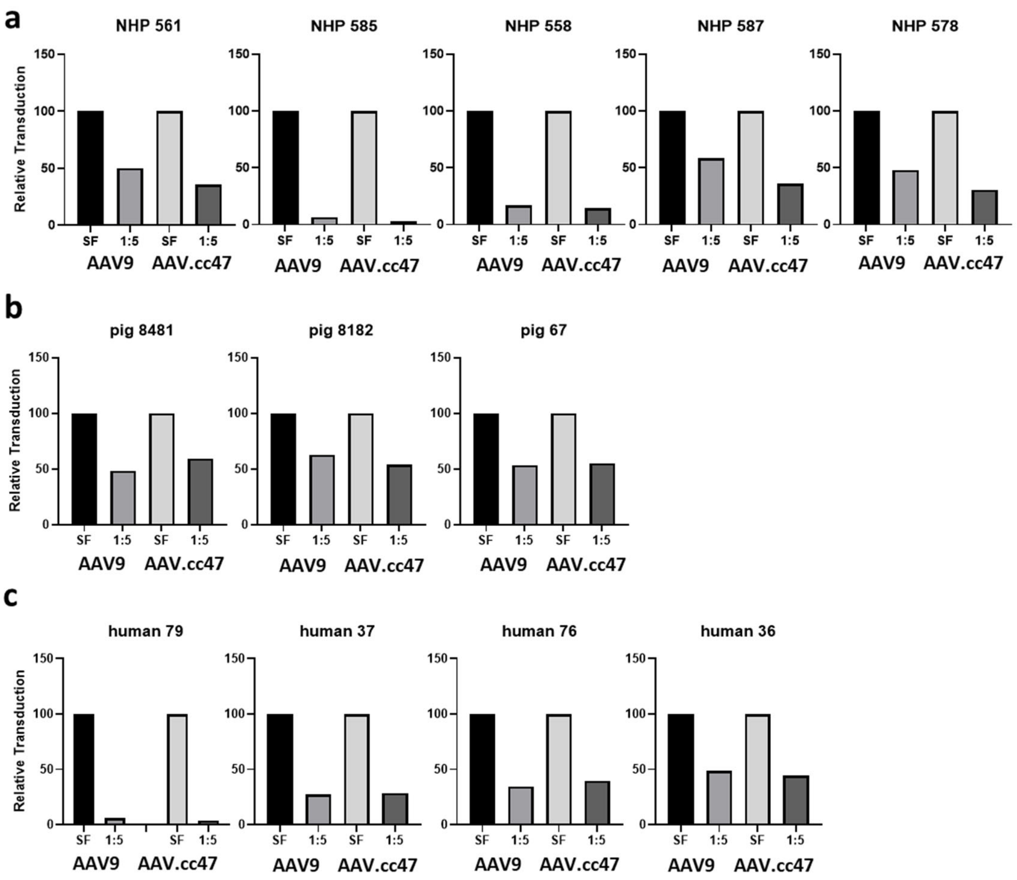

Supplementary Figure 14. Neutralizing antibody assay with serum from different species. AAV9 or AAV.cc47 vectors packaging a single stranded genome with the CBA promoter driving firefly luciferase were incubated with and without serum from NHPs (**a**), pigs (**b**), and humans (**c**). Following incubation with serum, HEK293s were seeded on top of the incubated vectors at 10,000 cells/well and incubated at 37C for 24 hours. Cells were then harvested 24 hours post transduction and protein lysates were prepared for a Luciferase assay. Source data are provided as a Source Data file.

Supplementary Table 1. List of primers used in this study

Primer table	
**Name**	**Sequence 5' to 3'**
Ai9 gRNA 1	GCTCTAGAGTCGCAGATCCTC
Ai9 gRNA 2	GACGAAGTTATATTAAGGGTT
Dmd gRNA 1	TACACTAACACGCATATTTG
Dmd gRNA 2	CATTGCATCCATGTCTGACT
ITR F	AACATGCTACGCAGAGAGGGAGTGG
ITR R	CATGAGACAAGGAACCCCTAGTGATGGAG
AAV9 lib amp F	AGCACGGTCCAGGTCTTCAC
AAV9 lib amp R	ATGTCAGTCTAGACCAAAGTTCAACTGAAACGAATTAAACGG
Cre F	TGACGGTGGGAGAATGTTAATC
Cre R	GCTACACCAGAGACGGAA
SV40 F	GCAGACATGATAAGATACATTGATGAGTT
SV40 R	AGCAATAGCATCACAAATTTCACAA
Ai9 gRNA1 qPCR F	GCTCTAGAGTCGCAGATCCTCGTTTTAG
Ai9 gRNA2 qPCR F	GACGAAGTTATATTAAGGGTTGTTTTAGTAC
common R qPCR	CGCCAACAAGTTGACGAGAT
SaCas9 F v1	GGAGCTTCATCCAGAGCATC
SaCas9 R v1	CGTCCTTGCTGTTCTTCTCC
SaCas9 F v2	AAAGGGTACAAGCACCATGC
SaCas9 R v2	TTCTTGGCTTTGTCCAGCTT
Actb qPCR F	CATTGCTGACAGGATGCAGAAGG
Actb qPCR R	TGCTGGAAGGTGGACAGTGAGG
CMV F	CAAGTACGCCCCCTATTGAC
CMV R	AAGTCCCGTTGATTTTGGTG
eGFP F	AGTGCTTCAGCCGCTACCC
eGFP R	GTTGTACTCCAGCTTGTGCC
Dmd gRNA1 qPCR F	TACACTAACACGCATATTTGGTTTT
Dmd gRNA2 qPCR F	TGCATCCATGTCTGACTGTT
Dmd exon 4-5 F	GGCACTGCGGGTCTTACA
Dmd exon 4-5 R	CATCCACTATGTCAGTGCTTCCTAT
Dmd exon 4-5 probe	/56-FAM/TTCACTAAA/ZEN/TCAACATTATTTTTC/3IABkFQ/
Dmd exon 22-24 F	CTGAATATGAAATAATGGAGGAGAGACTCG
Dmd exon 22-24 R	CTTCAGCCATCCATTTCTGTAAGGT
Dmd exon 22-24 probe	/56-FAM/ATGTGATTC/ZEN/TGTAATTTCC/3IABkFQ/
mCherry F	CCACCTACAAGGCCAAGAA
mCherry R	CTGTTCCACGATGGTGTAGT

Supplementary Table 2. Enrichment of AAV.cc47 following cycling in pigs, mice, and NHPs.

Capsid	Sequence	Parental %	Evolved %	Fold enrichment	Animal
AAV.cc47	GVSLGGG	0.00089	0.0011	1	Pig
AAV9	NGSGQNQ	0.99088	4.2210	4	
AAV.cc47	GVSLGGG	0.00089	0.1339	150	Mouse
AAV9	NGSGQNQ	0.99088	20.9090	21	
AAV.cc47	GVSLGGG	0.00089	61.5220	68934	NHP
AAV9	NGSGQNQ	0.99088	14.5160	15	
