# Supplementary material for: Cross-species evolution of a highly potent AAV variant for therapeutic gene transfer and genome editing
Source: Nat Commun. 2022 Oct 10;13:5947. doi: 10.1038/s41467-022-33745-4 (PMC9548504; doi:10.1038/s41467-022-33745-4)
Supplement: Supplementary file 3 — Reporting Summary [file 41467_2022_33745_MOESM3_ESM.pdf]

nature research

Corresponding author(s): Aravind Asokan

Last updated by author(s): 09/23/2022

Reporting Summary

Nature Research wishes to improve the reproducibility of the work that we publish. This form provides structure for consistency and transparency in reporting. For further information on Nature Research policies, see our Editorial Policies and the Editorial Policy Checklist.

Statistics

For all statistical analyses, confirm that the following items are present in the figure legend, table legend, main text, or Methods section.

n/a	Confirmed	
☐	☒	The exact sample size (*n*) for each experimental group/condition, given as a discrete number and unit of measurement
☐	☒	A statement on whether measurements were taken from distinct samples or whether the same sample was measured repeatedly
☐	☒	The statistical test(s) used AND whether they are one- or two-sided<br>*Only common tests should be described solely by name; describe more complex techniques in the Methods section.*
☒	☐	A description of all covariates tested
☐	☒	A description of any assumptions or corrections, such as tests of normality and adjustment for multiple comparisons
☐	☒	A full description of the statistical parameters including central tendency (e.g. means) or other basic estimates (e.g. regression coefficient) AND variation (e.g. standard deviation) or associated estimates of uncertainty (e.g. confidence intervals)
☐	☒	For null hypothesis testing, the test statistic (e.g. *F*, *t*, *r*) with confidence intervals, effect sizes, degrees of freedom and *P* value noted<br>*Give P values as exact values whenever suitable.*
☒	☐	For Bayesian analysis, information on the choice of priors and Markov chain Monte Carlo settings
☒	☐	For hierarchical and complex designs, identification of the appropriate level for tests and full reporting of outcomes
☐	☒	Estimates of effect sizes (e.g. Cohen's *d*, Pearson's *r*), indicating how they were calculated

Our web collection on statistics for biologists contains articles on many of the points above.

Software and code

Policy information about availability of computer code

Data collection	AAV9 monomer, trimer, and full capsid modeling was performed using PyMol 2.5.2. AAV variant detection by NGS and determination of fold enrichment were performed using 2 previously published in-house Perl scripts in Strawberry Perl for Windows v 5.32.1.1 software. The scripts have been deposited into Zenodo repository and can be accessed via DOI 10.5281/zenodo.7075694. Histological quantification was performed using ImageJ v 1.52a.
Data analysis	Graphs, statistical analyses, and heat map were generated using Prism software 9.3.1 (GraphPad). Bubble plots were generated using R studio v3.5.2 with ggplot package v 3.3.5. Consensus motif was determined using WebLogo v 2.8.2 software from University of California-Berkley.

For manuscripts utilizing custom algorithms or software that are central to the research but not yet described in published literature, software must be made available to editors and reviewers. We strongly encourage code deposition in a community repository (e.g. GitHub). See the Nature Research guidelines for submitting code & software for further information.

Data

Policy information about availability of data

All manuscripts must include a data availability statement. This statement should provide the following information, where applicable:

- Accession codes, unique identifiers, or web links for publicly available datasets
- A list of figures that have associated raw data
- A description of any restrictions on data availability

The NGS datasets for capsid libraries reported in this article are available under Sequence Read Archive accession code PRJNA869670. All other data associated with this study are present in the paper or the Supplementary Materials. Source data for each relevant figure is provided in a Source Data file. The data that support the findings of this study are available from the corresponding author upon reasonable request. Correspondence and requests for materials should be addressed to AA at aravind.asokan@duke.edu.

Field-specific reporting

Please select the one below that is the best fit for your research. If you are not sure, read the appropriate sections before making your selection.

- [x] Life sciences
- [ ] Behavioural & social sciences
- [ ] Ecological, evolutionary & environmental sciences

For a reference copy of the document with all sections, see nature.com/documents/nr-reporting-summary-flat.pdf

Life sciences study design

All studies must disclose on these points even when the disclosure is negative.

Sample size	Biological replicates (N=3-6 where applicable) and technical replicates (N=2) were chosen in order to generate means and standard error means that would allow various statistical analyses as outlined in the methods. No statistical calculation was performed to choose sample size. Sample sizes in this range have successfully been used by our laboratory for in vivo studies in the past. The max possible mice were used for each study based on breeding schedules and mouse availability at time of study.
Data exclusions	No data were excluded from analyses.
Replication	To confirm reproducibility for all data presented, each experiment and each assay was performed at least two times with no issues of reproducibility. For data where statistical analysis was utilized, the experiments were performed at least 2 times independently. In addition there were at least two technical replicates for each experiment.
Randomization	With the experiments used for this study, there was no need for randomization of our assays except for our therapeutic studies for Pompe disease and Duchenne muscular dystrophy. This is due in part to the multiple different reporter transgenes evaluated in vivo in this manuscript to assess the transduction efficiency of AAV.cc47. Since the Pompe and DMD studies were the only therapeutic evaluation for gene transfer and genome editing, respectively, these mice were chosen to be randomized for each.
Blinding	Blinding was not possible for studies except Pompe because the individual experiments were conducted and analyzed by a sole operator in our laboratory. The Pompe evaluation was performed with two operators from two separate laboratories so blinding of group allocation was performed during experiments and analysis. Only injections of test reagents in vivo were blinded in all relevant experiments.

Reporting for specific materials, systems and methods

We require information from authors about some types of materials, experimental systems and methods used in many studies. Here, indicate whether each material, system or method listed is relevant to your study. If you are not sure if a list item applies to your research, read the appropriate section before selecting a response.

Materials & experimental systems

n/a	Involved in the study	
	x	Antibodies
	x	Eukaryotic cell lines
x		Palaeontology and archaeology
	x	Animals and other organisms
x		Human research participants
x		Clinical data
x		Dual use research of concern

Methods

n/a	Involved in the study	
x		ChIP-seq
x		Flow cytometry
x		MRI-based neuroimaging

Antibodies

Antibodies used	rabbit polyclonal anti-RFP antibody (1:500; 600-401-379; Rockland), rabbit polyclonal anti-mCherry antibody (1:750, ab167453; Abcam), rabbit polyclonal anti-laminin antibody (1:300; ab11575; Abcam), mouse monoclonal anti-dystrophin antibody (1:500; D8168; Sigma-Aldrich), rabbit monoclonal anti-GFP antibody (1:750 dilution, G10362; Invitrogen), chicken polyclonal anti-mCherry antibody (1:750; ab205402; Abcam), rabbit monoclonal anti-NeuN antibody (1:500; EPR12763; Abcam), mouse monoclonal anti-NeuN antibody (1:500; ab104224 ; Abcam), rabbit monoclonal recombinant anti-GFP antibody (1:750; ab183735; Abcam), goat anti-rabbit Alexa Fluor 647 (1:1000, ab150079; Abcam), goat anti-rabbit Alexa Fluor 488 (1:500, A-11008; Invitrogen), goat anti-mouse Alexa Fluor 488 (1:500, A-11008; Invitrogen), goat anti-chicken Alexa Fluor 488 (1:500; A-11039; Invitrogen), goat anti-rabbit Alexa Fluor 647 (1:500; A-21245; Invitrogen), goat anti-mouse Alexa Fluor 647 (1:500; ab150115; Abcam).
Validation	rabbit polyclonal anti-RFP antibody, 1:500; 600-401-379; Rockland- https://www.rockland.com/categories/primary-antibodies/rfp-antibody-pre-adsorbed-600-401-379, PMID: #33202244 Russo, G. L., Sonsalla, G., Natarajan, et al., (2021). CRISPR-Mediated Induction of Neuron-Enriched Mitochondrial Proteins Boosts Direct Glia-to-Neuron Conversion. Cell stem cell, 28(3), 524–534.e7. https://doi.org/10.1016/j.stem.2020.10.015. Anti-RFP 600-401-379 antibody was confirmed by immunofluorescence staining of skeletal muscle sections in Garcia-Prat et al. (2020) Nat Cell Biol 22, 1307–1318.

rabbit polyclonal anti-mCherry antibody, 1:750, ab167453; Abcam- https://www.abcam.com/mcherry-antibody-ab167453.html, PMID: #33907215 Huerta-Ocampo, I., Dautan, D., Gut, N. K., et al., (2021). Whole-brain mapping of monosynaptic inputs to midbrain cholinergic neurons. Scientific reports, 11(1), 9055. https://doi.org/10.1038/s41598-021-88374-6. Anti-mCherry ab167453 antibody was confirmed by immunofluorescence staining in HEK293s cells transfected with a mCherry expressing vector (manufacture's website) and immunofluorescence staining of AAV transduced mouse brains (PMID #33907215).

rabbit polyclonal anti-laminin antibody, 1:300, ab11575; Abcam- https://www.abcam.com/laminin-antibody-ab11575.html, PMID# 33845891 Chen, M., Shi, H., Gou, S., et al. (2021). In vivo genome editing in mouse restores dystrophin expression in Duchenne muscular dystrophy patient muscle fibers. Genome medicine, 13(1), 57. https://doi.org/10.1186/s13073-021-00876-0. Anti-laminin antibody ab11575 was confirmed by immunofluorescence staining of human tongue sections (manufacture's website) and immunofluorescence staining of AAV transduced mouse skeletal muscles (PMID# 33845891).

mouse monoclonal anti-dystrophin antibody clone MANDYS8, 1:500, D8168; Sigma-Aldrich- https://www.sigmaaldrich.com/US/en/product/sigma/d8168, PMID# 30778238 Nelson, C. E., Wu, Y., Gemberling, M. P., et al. (2019). Long-term evaluation of AAV-CRISPR genome editing for Duchenne muscular dystrophy. Nature medicine, 25(3), 427–432. https://doi.org/10.1038/s41591-019-0344-3. Anti-dystrophin antibody D8168 was confirmed by immunofluorescence staining of human tongue sections (manufacture's website) and immunofluorescence staining of AAV transduced mouse skeletal muscles (PMID# 30778238).

rabbit monoclonal anti-GFP antibody, 1:750, G10362; Invitrogen- https://www.thermofisher.com/antibody/product/GFP-Antibody-Recombinant-Monoclonal/G10362, PMID# 21685891 Calvo, F., Sanz-Moreno, V., Agudo-Ibáñez, L., et al., (2011). RasGRF suppresses Cdc42-mediated tumour cell movement, cytoskeletal dynamics and transformation. Nature cell biology, 13(7), 819–826. https://doi.org/10.1038/ncb2271. Anti-GFP antibody G10362 was confirmed by immunofluorescence staining of HEK293s cells transfected with a eGFP expressing vector (manufacture's website) and immunofluorescence staining of AAV transduced mouse brains (PMID #27699236).

mouse monoclonal anti-NeuN antibody. 1:500, ab104224; Abcam- https://www.abcam.com/neun-antibody-1b7-neuronal-marker-ab104224.html, PMID #30358470 Gong, Y., Berenson, A., Laheji, F., et al., (2019) Intrathecal Adeno-Associated Viral Vector-Mediated Gene Delivery for Adrenomyeloneuropathy. Human Gene Therapy, 30(5): 544-555. https://doi.org/10.1089/hum.2018.079. Anti-NeuN antibody was confirmed by immunofluorescence staining of mouse brain sections from mice injected with AAV delivering GFP.

rabbit polyclonal anti-NeuN antibody. 1:500, EPR12763, Abcam- https://www.abcam.com/neun-antibody-epr12763-neuronal-marker-ab177487.html, PMID 35474956 Palfi, A., Chadderton, N., Millington-Ward, S., et al., (2022) AAV-PHP.eB transduces both the inner and outer retina with high efficacy in mice. Molecular Therapy Methods and Clinical Development, 25:236-249. https://doi.org/10.1016/j.omtm.2022.03.016. Anti-NeuN antibody was confirmed by immunofluorescence staining of mouse retinal ganglion cells from mice injected with AAV delivering GFP.

chicken polyclonal anti-mCherry antibody. 1:750, ab205402, Abcam- https://www.abcam.com/mcherry-antibody-ab205402.html, PMID 34318750, Xie, Z., Wang, M., Liu, Z., et al., (2021) Transcriptomic encoding of sensorimotor transformation in the midbrain. eLife, 10: e69825. https://doi.org/10.7554/eLife.69825. Anti-mCherry antibody was confirmed by immunofluorescence staining of mouse brains injected with AAV delivering mCherry.

rabbit monoclonal recombinant anti-GFP antibody. 1:750, ab183735, Abcam- https://www.abcam.com/gfp-antibody-epr14104-89-ab183735.html, PMID 31666602, Xiao, D., Bi, R., Liu, X., et al., (2019) Notch Signaling Regulates MMP-13 Expression via Runx2 in Chondrocytes. Sci Rep, 9(1):15596. https://doi: 10.1038/s41598-019-52125-5. Anti-GFP antibody was confirmed by immunohisyochemistry staining of chondrocytes in vivo.

Eukaryotic cell lines

Policy information about cell lines

Cell line source(s)	HEK293s are from UNC Vector Core, Huh7 scramble and Huh7 AAVR knockout cells were produced in our laboratory at Duke University.
Authentication	HEK293 and Huh7 scramble cells were not authenticated. Huh7 AAVR knockout cells were confirmed by both western blot and next generation sequencing to confirm loss of AAVR protein and deletion of AAVR from the genome, respectively.
Mycoplasma contamination	HEK293s, Huh7 scramble, and Huh7 AAVR knockout cells tested negative for mycoplasma contamination.
Commonly misidentified lines (See ICLAC register)	No commonly misidentified cell lines were used in this study.

Animals and other organisms

Policy information about studies involving animals; ARRIVE guidelines recommended for reporting animal research

Laboratory animals	All mouse (mus musculus) IV dosing studies occurred in adult male and female mice aged 8-10 weeks at time of dosing. Strains include: wild-type C57/Bl6 mice, Ai9 reporter mice, Gaa knockout mice, and mdx;nGFP;Pax7 mice. All mouse (mus musculus) ICV dosing studies occurred in male and female C57/Bl6 mice post-natal day 0-1 at time of dosing. All mouse strains used in this study were maintained at Duke University School of Medicine with the assistance of Duke's Division of Laboratory Animal Resources (DLAR). Mice were housed in a temperature-controlled (~18-23C, 40-60% humidity) and enriched environment, with a 12 hour light/dark cycle, and provided standard chow and water. All monkey ICM dosing studies occurred in adult male and female 2 year old Cynomolgus macaques at time of dosing. All pig IT dosing studies occurred in male 4 week old pigs at time of dosing. The strain

	included a cross of Landrace, Yorkshire, and Duroc strains.
Wild animals	Study did not include wild animals.
Field-collected samples	Study did not include samples collected from the field.
Ethics oversight	All mouse and pig protocols were approved by the Institutional Animal Care and Use Committee (IACUC) at Duke University (mouse; Protocol A189-21-09) and North Carolina State College of Veterinary Medicine (pigs; Protocol 20-425). NHP studies and protocols were reviewed and approved by IACUC at Southern Research (Birmingham, AL; Protocol 15863.01) and CR-MWN IACUC at Charles River Labs (Kalamazoo, MI; Protocol ID: 2728-017), both accredited by the Association for Assessment and Accreditation of Laboratory Animal Care-International (AAALAC). All studies were carried out using applicable Standard Operating Procedures at Southern Research (Birmingham, AL) and Charles River Labs (Kalamazoo, MI) and previously reported by our lab (37). During the study, the care and use of animals was conducted in accordance with the guidelines of the USA National Research Council, the US Department of Agriculture (Animal Welfare Act; Public Law 99-198) and those of the Guide for the Care and Use of Laboratory Animals (National Academies Press, 2011).

Note that full information on the approval of the study protocol must also be provided in the manuscript.
